# Oxidative Stress Causes Masculinization of Genetically Female Medaka Without Elevating Cortisol

**DOI:** 10.3389/fendo.2022.878286

**Published:** 2022-06-16

**Authors:** Koki Mukai, Seiji Hara, Konosuke Sakima, Ryo Nozu, Takashi Yazawa, Takeshi Kitano

**Affiliations:** ^1^ Department of Biological Sciences, Graduate School of Science and Technology, Kumamoto University, Kumamoto, Japan; ^2^ Department of Biochemistry, Asahikawa Medical University, Asahikawa, Japan; ^3^ International Research Center for Agricultural and Environmental Biology, Graduate School of Science and Technology, Kumamoto University, Kumamoto, Japan

**Keywords:** oxidative stress, cortisol, masculinization, environmental sex determination, medaka Oryzias latipes

## Abstract

Medaka (*Oryzias latipes*) is a teleost fish with an XX/XY sex determination system. Sex reversal from female-to-male (masculinization of XX fish) can be induced through cortisol elevation from exposure to environmental stress such as high temperature during sexual differentiation. However, the effects of oxidative stress, generated *via* metabolic reactions and biological defense mechanisms, on the sexual differentiation of medaka are unclear. Here, we investigated the effect of oxidative stress on medaka sexual differentiation using hydrogen peroxide (H_2_O_2_), which induces oxidative stress in vertebrates. H_2_O_2_ treatment from 0 to 5 days post-hatching induced masculinization of wild-type XX medaka, but not of gonadal soma-derived growth factor (*gsdf*) or peroxisome proliferator-activated receptor alpha-a (*pparaa*) knockout XX fish. Co-treatment with an oxidative stress inhibitor caused masculinization recovery but co-treatment with a cortisol synthesis inhibitor did not. H_2_O_2_ treatment significantly upregulated *gsdf* and *pparaa* expression in XX medaka. However, H_2_O_2_ did not elevate cortisol levels in medaka larvae during sexual differentiation. These results strongly indicate that oxidative stress induces masculinization of XX medaka without causing elevation of cortisol.

## Introduction

In the offspring of many vertebrates sex is determined genetically through sex chromosomes inherited from their parents—with parthenogenesis being the exception (1–3). Sex determination systems differ across species. For example, most mammals exhibit a male heterozygous XX/XY system, and birds and some reptiles exhibit a female heterozygous ZZ/ZW system. Sex determination can also be influenced by environmental stresses (e.g., temperature and pH) during the sexual differentiation period in some reptiles (4, 5), amphibians (6, 7), and fish (8, 9). However, the molecular mechanisms underlying environmental sex determination in these species are poorly understood.

Medaka (*Oryzias latipes*) is commonly used as a model laboratory organism because it has a short generation interval, a small genome size, and is easy to handle and rear. *dmy/dmrt1bY*, the medaka sex-determining gene located on the Y chromosome, has been identified (10, 11). Medaka also make an excellent vertebrate model organism for molecular biology and genetic experiments because transgenic techniques and gene knockout (KO) systems using transcription activator-like effector nuclease (TALEN), or clustered regularly interspaced short palindromic repeat (CRISPR)/CRISPR-associated protein 9 (Cas9) have been established in this species (12–15).

Exposure to high temperature (HT) causes female-to-male sex reversal (masculinization) of medaka during the sexual differentiation period (16–18). Exposure to HT elevates cortisol levels and increases expression of gonadal soma-derived growth factor (*gsdf*), a TGF-beta superfamily gene related to testis differentiation in teleosts, and decreases the expression of *cyp19a1a*, which encodes cytochrome P450 aromatase, an ovary differentiation factor (19). Therefore, it was concluded that HT promotes male development and suppresses female development. Moreover, it was recently reported that peroxisome proliferator-activated receptor alpha-a (*pparaa*), which regulates the expression of fatty acid-related genes, is activated by HT and cortisol and its activation leads to the masculinization of XX medaka (20). Recently, it was reported that lipid metabolism may regulate female-to-male sex reversal in starved medaka (21). Hence, changes in the lipid metabolism system, induced as a stress response, may also be involved in the masculinization of XX medaka.

Reactive oxygen species (ROS) generated by metabolic reactions (e.g., fatty acid oxidation and the mitochondrial respiratory chain) have a physiological function in intercellular signaling, but excessive ROS cause oxidative damage in various cellular molecules (e.g., DNA, RNA, proteins, and lipids) (22–26). For example, oxidative stress from excessive ROS has been linked to diseases such as alcoholic liver disease (27–29). Zhang et al. (2016) reported that inorganic mercury (Hg) exposure caused oxidative stress and histological damage in the gonads of adult zebrafish, and altered sex hormone levels by disrupting the transcription of genes involved in the hypothalamic-pituitary-gonadal axis. (30). However, the effect of oxidative stress on sexual differentiation was not examined.

In the present study, we investigated the effect of oxidative stress on the sexual differentiation of medaka using hydrogen peroxide (H_2_O_2_), which induces oxidative stress in vertebrates (31). We then assessed sex-reversal ratio and fertility in the adults, and investigated cortisol levels and expression patterns of sex-related genes in medaka larvae. Finally, we studied the relationship between masculinization by oxidative stress and GSDF or PPARα function using *gsdf* and *pparaa* KO medaka.

## Materials and Methods

### Ethics Statement

This study was performed using protocols approved by the Animal Care and Use Committee of Kumamoto University (A2020-014). All methods were carried out in accordance with the relevant guidelines and regulations. The study was performed according to the ARRIVE guidelines along with the general guidelines and ethical approval.

### Animals

Wild-type, *pparaa* KO (20), and *gsdf* KO lines (32) originating from an FLFII medaka line (33) were used in this study. *pparaa* KO and *gsdf* KO lines were generated using the CRISPR/Cas9 system and maintained by brother-sister mating of the genetically homogenous medaka (20, 32). *pparaa* KO and *gsdf* KO XX medaka do not masculinize with cortisol treatment (20, 32), while some *gsdf* KO but not *pparaa* KO XY fish become females having ovaries under normal conditions, while others become normal males having testes (34). Fish embryos and larvae were maintained in embryo-rearing medium (ERM: 17 mM NaCl, 0.4 mM KCl, 0.27 mM CaCl_2_.2H_2_O, 0.66 mM MgSO_4_, pH 7) at 26°C under a 14-h light and 10-h dark cycle.

### Experimental Treatment

To confirm the masculinization of XX medaka by oxidative stress, H_2_O_2_ treatments were performed with 0.75 and 2 mM H_2_O_2_ (purity 30%, CAS RN: 7722-84-1; Wako Pure Chemical, Osaka, Japan) using wild-type larvae from 0 to 5 days post-hatching (dph) in 6-well culture plates (Corning, Glendale, AZ) with the water being changed daily. A rescue test to reduce oxidative stress through antioxidant supplementation was conducted with either 1 or 10 μM N-acetyl-L-cysteine (NAC; Wako) dissolved in Dimethyl sulfoxide (DMSO; Sigma-Aldrich, Saint Louis, MO) as previously described (20), using larvae from 0 to 5 dph in 6-well culture plates with the water being changed daily.

To investigate whether other XX medaka lines undergo masculinization from oxidative stress, *pparaa* KO, and *gsdf* KO medaka larvae were treated with 2 mM H_2_O_2_ under the conditions outlined above. Finally, treatment with 5 μM metyrapone (Sigma-Aldrich) dissolved in ethanol (Wako) as previously described (17), was used in conjunction with 2 mM H_2_O_2_ in FLFII medaka larvae to investigate the relationship between cortisol and oxidative stress-induced masculinization. The survival rates and the body sizes in adults are shown in **Supplementary Tables S1**, **S2**, respectively.

### Genetic Sexing

The genetic sex of the adults (about 2 months old) was determined by genomic PCR. The PCR was performed using specific primers for *dmy*/*dmrt1bY* as previously described (17). PCR conditions were as follows: preheating at 95°C for 10 min, 40 cycles of 94°C for 30 sec, 59°C for 30 sec, 72°C for 1 min, and a final extension at 72°C for 5 min. The genetic sex of the larvae at 5 dph was determined by the appearance of leucophores as previously described (33).

### Histological Analysis of Gonads

Histological analysis was performed as previously described (17). Adult fish were prepared for examining the gonadal phenotype and calculating sex ratios. The tissue was fixed in Bouin’s solution at 4°C overnight, embedded in paraffin, sectioned serially at a thickness of 5 μm, stained with hematoxylin and eosin, and then imaged with an Eclipse Ci-E microscope (Nikon, Tokyo, Japan).

### Fertility Assessment

The fertility of adult XX male medaka (approximately 2 months old) after H_2_O_2_ treatment from 0 to 5 dph was assessed by natural mating with fertile partners (wild-type XX female medaka). A minimum of 80 eggs were analyzed to determine the fertilization rate. Adult XY male medaka (approximately 2 months old) without H_2_O_2_ treatment were used as the control group. Fertility assessment was conducted with three mating pairs.

### Quantitative Real-Time PCR

Gene expression analysis by quantitative real-time PCR was used to further investigate the mechanism of XX medaka masculinization by oxidative stress. Total RNA was extracted from the gonad regions of wild-type larvae (N = 4, 10 pooled samples) treated with or without 2 mM H_2_O_2_ from 0 to 5 dph using ISOGEN (Nippon Gene, Tokyo, Japan) as previously described (19). Briefly, reverse transcription was performed at 37°C for 30 min using a ReverTra Ace^®^ qPCR RT Master Mix (Toyobo, Osaka, Japan). Quantitative real-time PCR was performed with specific primers (**Supplementary Table S3**) on a LightCycler 480 (Roche, Mannheim, Germany) using SYBR Green I Master Mix (Roche). The PCR conditions were as follows: 95°C for 5 min, then 45 cycles of 95°C for 5 min, 59°C for 10 s, and 72°C for 10 s. Relative gene expression levels were calculated using a delta-CT method. The RefFinder tool, which integrates four specific algorithms [GeNorm (35), NormFinder (36), BestKeeper (37), and the comparative delta-Ct method (38)], was used for the assessment and screening of three candidate reference genes [*elongation factor 1 alpha* (*ef1α*), *β-actin*, and *glyceraldehyde-3-phosphate dehydrogenase* (*gapdh*)]. *ef1a* was the most stably expressed gene and was used as a reference gene (**Supplementary Table S4**).

### Cortisol Measurement

To confirm the stress level in each treatment, a cortisol measurement was performed. Wild-type medaka larvae were treated with or without 2 mM H_2_O_2_ from 0 to 5 dph. Metyrapone (5 μM) was added to the 2 mM H_2_O_2_ treatment group and cortisol (5 μM) treatment was used as a positive control.

Steroid hormones were extracted as previously described (39). Briefly, five pooled larvae were homogenized in phosphate-buffered saline (137 mM NaCl, 2.68 mM KCl, 8.1 mM Na_2_HPO_4_, 1.47 mM KH_2_PO_4_; pH 7.4). Steroids were extracted three times from the homogenates into diethyl ether according to previous methods used for teleosts (40–42), and cortisol levels were measured using a cortisol EIA kit (Cayman Chemical, Ann Arbor, MI) according to the manufacturer’s instructions. Individual cortisol levels (per fish) were determined by dividing the average measurement by the number of pooled samples.

### Statistical Analysis

Statistical analysis was performed using Statcel 3 with Excel (OMS, Saitama, Japan). Significant differences in sex-reversal ratio among the treatments were determined by the chi-squared test. Student’s t-test was used to detect significant differences in fertility. One-way ANOVA, Tukey-Kramer, and Scheffe’s F tests were used to statistically analyze the gene expression and cortisol measurement data.

## Results

### The Effect of Oxidative Stress on Sex Differentiation of XX Medaka

**Figure 1:**
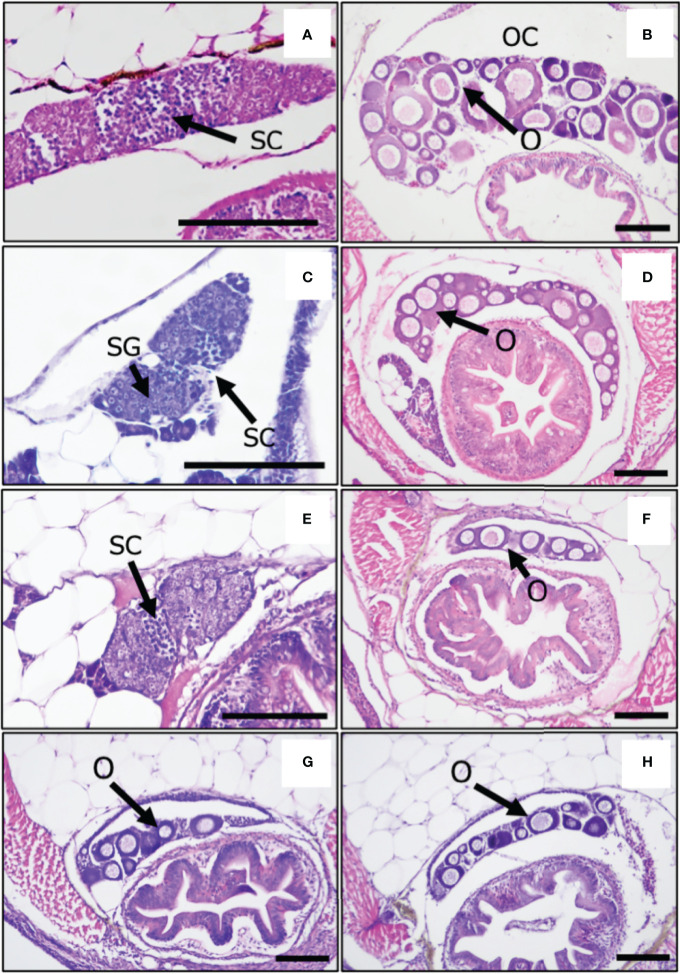
Gonads of adult medaka at 2 months post-hatching. **(A)** XY male, control, **(B)** XX female, control, **(C)** sex-reversal XX male, **(D)** no sex-reversal XX female with H_2_O_2_ treatment, **(E)** H_2_O_2_ + metyrapone, **(F)** H_2_O_2_ + NAC, **(G)** *gsdf* KO XX female, **(H)** *pparaa* KO XX female with H_2_O_2_ treatment. SC, spermatocytes; SG, spermatogonia; O, oocytes; OC, ovarian cavities; Scale bars: 100 μm.

To investigate whether oxidative stress causes the masculinization of XX medaka, we treated medaka larvae with H_2_O_2_ from 0 to 5 dph, which is the shortest temperature-sensitive period determined by our previous study (17, 19). The sex-reversal ratio (at approximately 2 months of age) for each treatment is shown in **Table 1**. H_2_O_2_ treatment (0.75 mM H_2_O_2_; 14.3%, 2 mM H_2_O_2_; 35%) caused the masculinization of wild-type XX medaka in a concentration-dependent manner. Histological analysis showed that control XY males had normal testes with productive spermatogenesis **Figure 1A**) and XX female medaka had normal ovaries with young oocytes (**Figure 1B**). H_2_O_2_-treated, sex-reversed XX individuals had normal testes that included spermatocytes (**Figure 1C**), while other H_2_O_2_-treated XX individuals had normal ovaries, as in control females (**Figure 1D**). This was also observed after treatment with 2 mM H_2_O_2_ and metyrapone, a cortisol synthesis inhibitor (**Figure 1E**). Treatment with 2 mM H_2_O_2_ and NAC, an oxidative stress inhibitor, produced less masculinization (15.4%) than with 2 mM H_2_O_2_ alone (**Figure 1F**). No masculinization was observed in the *gsdf* or *pparaa* KO XX medaka treated with 2 mM H_2_O_2_ (**Figures 1G, H**), similar to the untreated *gsdf* and *pparaa* KO XX medaka (20, 32). There was a significant difference in sex-reversal ratio between XX controls and XX individuals treated with 2 mM H_2_O_2_ or XX individuals treated with 2 mM H_2_O_2_ and metyrapone (**Table 1**).

**Table 1:** Sex ratios in adult medaka. a and b: significant difference (p < 0.05).

No. of adult fish						
Genotype	Treatment	XY♂	XY♀	XX♂	XX♀	% of XX sex-reversal
wild-type	Control	10	0	0	11	0^a^
	0.75 mM H_2_O_2_	21	0	3	18	14.3^a^
	2 mM H_2_O_2_	17	0	7	13	35.0^b^
	2 mM H_2_O_2_ +Metyrapone	11	0	4	9	30.8^b^
	2 mM H_2_O_2_ + 1 µM NAC	16	0	2	11	15.4^a^
	2 mM H_2_O_2_ + 10 µM NAC	17	0	2	11	15.4^a^
*gsdf ^-/-^*	Control	7	0	0	7	0^a^
	2 mM H_2_O_2_	20	0	0	11	0^a^
*pparaa ^-/-^*	Control	10	0	0	8	0^a^
	2 mM H_2_O_2_	13	0	0	20	0^a^

**Figure 2:**
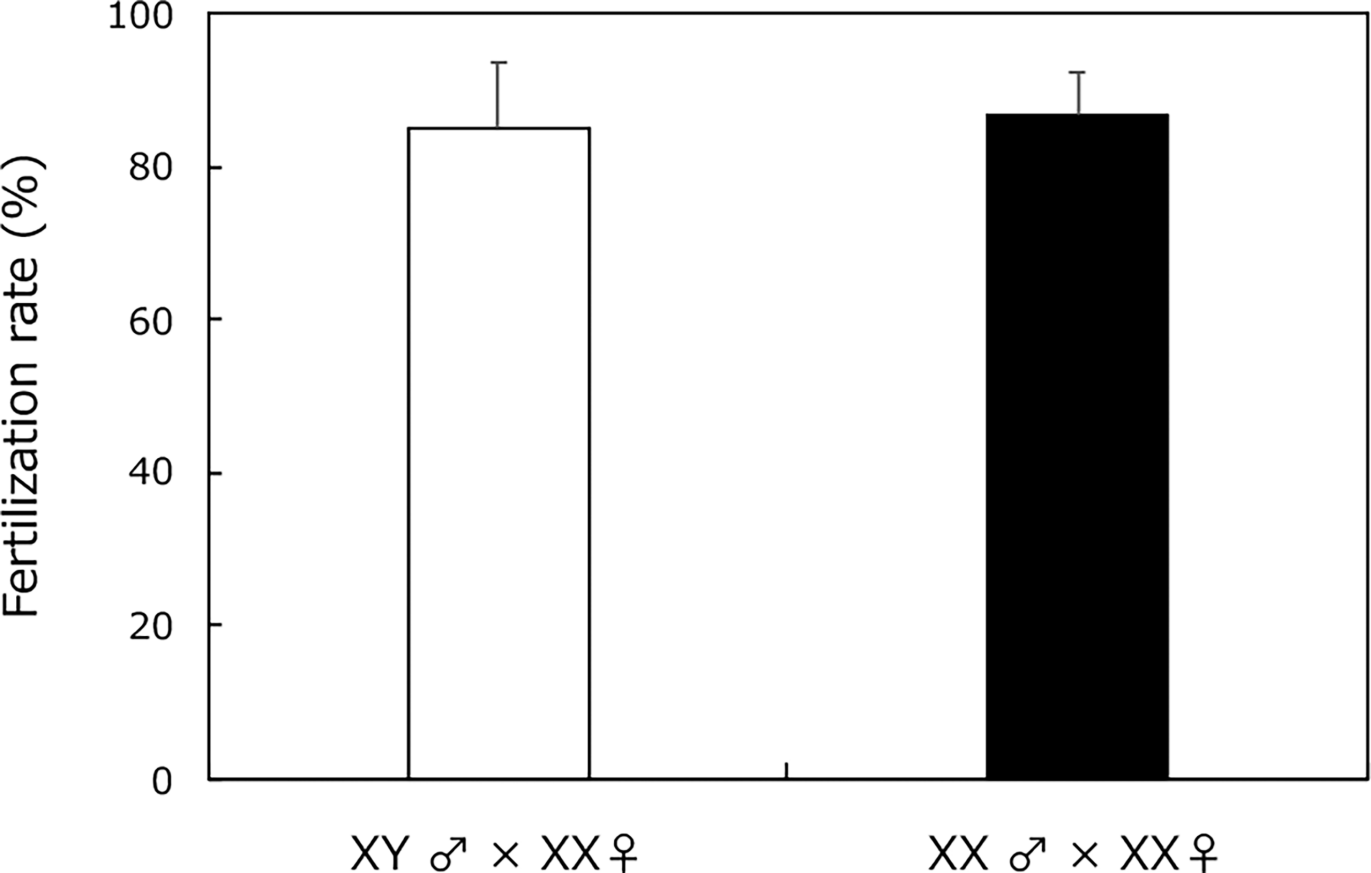
Fertility assessment of adult XY male without H_2_O_2_ treatment (white box) and sex-reversal XX male medaka with H_2_O_2_ treatment from 0 to 5 dph (black box) using natural mating with fertile partners (XX female medaka). Vertical bar: mean ± standard error of triplicate samples.

Wild-type XY males and sex-reversal XX males (from H_2_O_2_ treatment) had similar fertilization rates after mating with XX females (**Figure 2**). Every fertilized egg developed into a normal larva.

### Effects of Oxidative Stress on Gene Expression During Sex Differentiation

**Figure 3:**
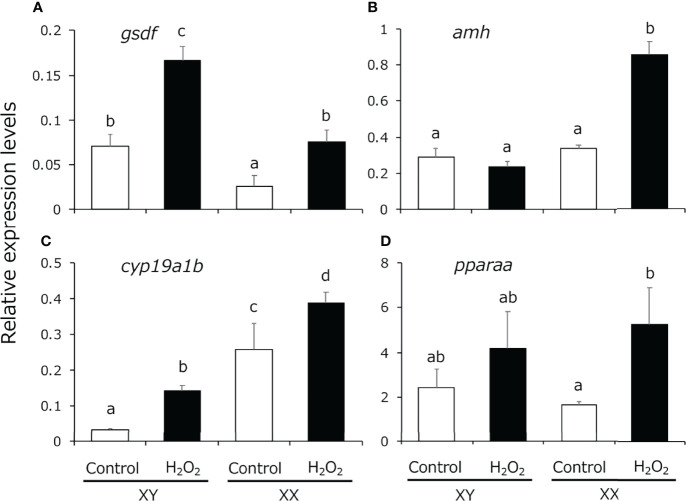
Quantitative real-time PCR analysis of the expression of **(A)** *gsdf*, **(B)** *amh*, **(C)** *cyp19a1b* and **(D)** *pparaa* in the gonadal region of controls (white box) and H_2_O_2_-treated medaka (black box) at 5 dph. Relative expression levels of the target genes were normalized to that of *ef1α*. Vertical bar: mean ± standard error of quadruplicate samples; a, b, c, d, and ab: significant difference (p < 0.05).

We analyzed the expression pattern of male-related genes [*gsdf* and *anti-Müllerian hormone* (*amh*)], female-related genes (*cyp19a1b*), which is expressed in the larval gonads and regulated by cortisol (20), and *pparaa* in 5-dph larvae exposed to H_2_O_2_-induced oxidative stress using quantitative real-time PCR. *gsdf e*xpression levels were significantly higher in XY medaka than in XX fish and the expression was increased in both sexes by H_2_O_2_ treatment (**Figure 3A**). *amh* expression was detected in both sexes to the same extent, similar to the findings in a previous study (43), and the expression in XX fish was significantly increased by H_2_O_2_ treatment (**Figure 3B**). *cyp19a1b* expression levels were significantly higher in XX medaka than in XY fish and the expression was significantly increased in both sexes by H_2_O_2_ treatment (**Figure 3C**). *pparaa* expression levels were detected in both sexes to the same extent and the expression in XX fish was significantly increased by H_2_O_2_ treatment (**Figure 3D**).

### Change of Cortisol Levels in Medaka Exposed to Oxidative Stress

**Figure 4:**
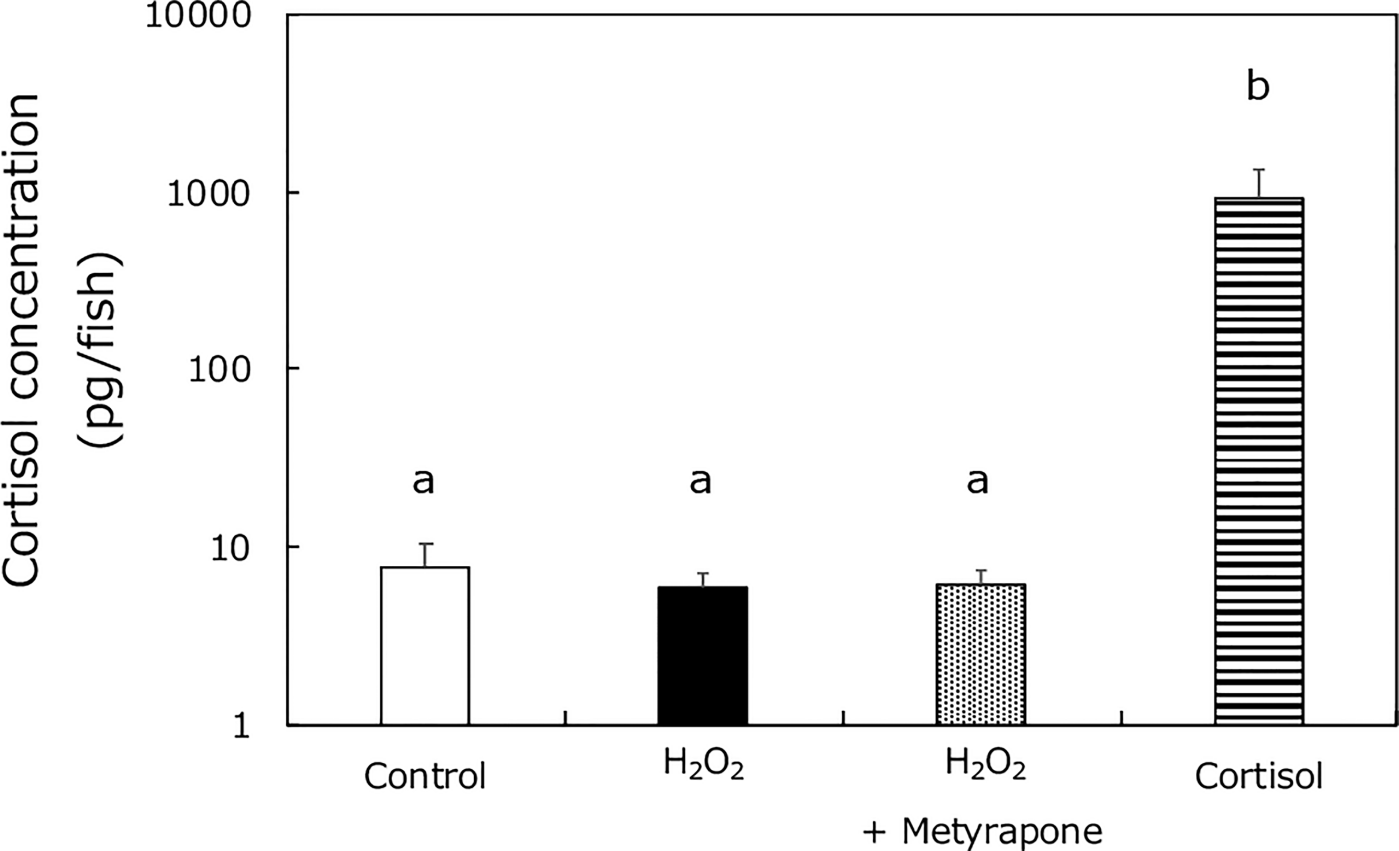
Cortisol levels in medaka larvae at 5 dph. Control (white box), H_2_O_2_ (black box), H_2_O_2_ with metyrapone (dotted box), and cortisol treatment (stripe box) groups. Vertical bar: mean ± standard error of quadruplicate samples; a and b: significant difference (p < 0.01).

To investigate if oxidative stress caused cortisol elevation in XX medaka (in a similar way to high temperature treatment), we assessed cortisol levels in wild-type medaka treated with: 0 or 2 mM H_2_O_2_, metyrapone, or cortisol, from 0 to 5 dph (**Figure 4**). The cortisol levels were similar in the H_2_O_2_-, and metyrapone-treated groups, in contrast to those in HT-treated medaka (17). Significant differences were detected between the cortisol treated group and the other groups.

## Discussion

To assess the effect of oxidative stress on sex differentiation of medaka, we analyzed histological changes of the gonad, cortisol levels, fertility rate, and sex-related gene expression using adults or larvae treated with H_2_O_2_ from 0 to 5 dph. Previous studies have shown that cortisol and HT treatments were more effective during the 5 days after hatching than before hatching (17, 20), although sexually dimorphic proliferation of germ cells has occurred by the hatching stage (44). Therefore, in this study, we treated larvae with H_2_O_2_ for 5 days after hatching. Our results indicate that oxidative stress causes masculinization of XX medaka. To our knowledge, this is the first report to show that oxidative stress can cause masculinization of a vertebrate species. Recently, it was reported that in a teleost, Atherinopsidae, whose sex can be determined by temperature, masculinization by continuous illumination was accompanied by significant increases in the expression of the stress axis activation gene, *crf*, and ROS antagonist effector genes, *gsr* and *cat*, indicating that both the stress axis and ROS response mechanisms are activated at this time (45). Therefore, although the masculinizing effect of oxidative stress in other species remains uncertain, there may be a link between continuous light, oxidative stress, and environmental sex determination in vertebrates.

The fertilization rate was similar between matings of H_2_O_2_-treated, sex-reversed XX males with XX females, and of control XY males with XX females. Moreover, the fertilized eggs developed into normal individuals. A previous study reported that Hg exposure induced oxidative stress in adult gonads and caused alterations in gonadal histology, sex hormone production, and sex-related gene expression in zebrafish (30). Additionally, atretic oocytes, and a loss of contact between the oocyte cell membrane and the follicular cell layer was observed. While the fertilization rate was not confirmed, the structure of the gonads was adversely affected and it was proposed that fertility would be disrupted (30). In the present study, we found that XX males, induced by oxidative stress from H_2_O_2_ treatment, exhibited normal fertility, which indicates that sex reversal by H_2_O_2_ treatment resulted in a normal masculinization cascade and had no toxic effects.

Previously, we showed that masculinization of XX medaka was caused by elevated cortisol levels because masculinization by HT was inhibited by treatment with metyrapone, an inhibitor of cortisol synthesis (17). Although metyrapone completely inhibits cortisol synthesis in medaka embryos (17), this drug did not decrease cortisol levels of fish larvae after hatching in this study. Therefore, higher concentrations of metyrapone may be needed to block completely cortisol synthesis in the larvae. However, masculinization of XX medaka by H_2_O_2_ treatment was not suppressed by treatment with metyrapone and no increase in cortisol levels was observed in the H_2_O_2_-treated group. These results strongly indicate that oxidative stress causes masculinization of XX medaka without elevating cortisol. Moreover, we found that H_2_O_2_ treatment dose-dependently induced masculinization of XX medaka but not all fish were masculinized, similar to cortisol-induced masculinization (16–18, 46). Some studies have been reported that cortisol induces oxidative stress in teleosts (47, 48). Taken together, these results strongly suggest that oxidative stress may act downstream of cortisol signaling in the process of stress-induced masculinization, although further investigation is needed.

In our previous studies, *pparaa* or *gsdf* KO XX medaka were not masculinized by cortisol treatment, which strongly indicated that *pparaa* and *gsdf* are involved in masculinization of XX medaka (20, 32). Consistently, in this study, masculinization of XX medaka by H_2_O_2_ treatment was completely suppressed in *pparaa* KO and *gsdf* KO fish, suggesting that masculinization of XX medaka by oxidative stress also occurred *via* PPARα and GSDF function. The *gsdf* is predominantly expressed in Sertoli cells and granulosa cells in mature medaka gonads (49). Moreover, deletion of *gsdf* results in phenotypic sex reversal of males to females (34), while *gsdf* transgenic XX medaka show masculinization (50). PPARα acts as a transcription factor that regulates the expression of genes related to fatty acid oxidation (51, 52). Hara et al. (2020) revealed that agonist-activation of PPARα induces the masculinization of XX medaka whereas treatment of *pparaa* KO medaka with cortisol or the agonist did not induce masculinization of XX medaka (20). In the present study, the expression levels of *gsdf* and *pparaa* were significantly elevated in XX larvae by H_2_O_2_ treatment. Surprisingly, the expression of *amh*, which is detected to the same extent in both sexes (17), and *cyp19a1b*, which is more highly expressed in XX individuals than in XY individuals (20), were also significantly induced in XX larvae by H_2_O_2_ treatment. Since molecular mechanisms and implications that oxidative stress induces the expression of these genes remain unclear at this time, it will be necessary to analyze them using *amh* and *cyp19a1b* KO medaka in the future. Thus, oxidative stress appears to cause masculinization of XX medaka by regulating the expression of these sex-related genes during the sex differentiation period.

Although this study shows that oxidative stress induces masculinization of XX medaka, the molecular pathway of stress-mediated masculinization was not fully elucidated. Previously, it has been reported that ROS interact with low-density lipoproteins to activate PPARα and subsequently limit inflammation, as indicated by PPAR-dependent repression of inducible nitric oxide synthase gene transcription (53). Therefore, oxidative stress is likely to induce masculinization of XX medaka through activation of PPARα, which in turn causes induction of the expression of male-related genes. Future studies will focus on the molecular pathway of masculinization mediated by stress.

In summary, H_2_O_2_ treatment induced masculinization of wild-type XX medaka but not of *gsdf* or *pparaa* KO XX fish. The masculinization could be prevented by co-treatment with the oxidative stress inhibitor, NAC, but not with the cortisol synthesis inhibitor, metyrapone. Moreover, H_2_O_2_ treatment significantly upregulated *gsdf* and *pparaa* expression in XX medaka. Notably, H_2_O_2_ did not elevate cortisol levels in medaka larvae during sexual differentiation. These results strongly indicate that oxidative stress induces masculinization of XX medaka without causing elevation of cortisol.

## Data Availability Statement

The original contributions presented in the study are included in the article/**Supplementary Material**. Further inquiries can be directed to the corresponding author.

## Ethics Statement

The animal study was reviewed and approved by the Animal Care and Use Committee of Kumamoto University (A2020-014). Written informed consent was obtained from the owners for the participation of their animals in this study.

## Author Contributions

TK obtained funding and designed the study. KM, SH, KS, RN and TY performed the experiments and collected the data. KM, RN and TK wrote the manuscript. All authors contributed to the article and approved the submitted version.

## Conflict of Interest

The authors declare that the research was conducted in the absence of any commercial or financial relationships that could be construed as a potential conflict of interest.

## Publisher’s Note

All claims expressed in this article are solely those of the authors and do not necessarily represent those of their affiliated organizations, or those of the publisher, the editors and the reviewers. Any product that may be evaluated in this article, or claim that may be made by its manufacturer, is not guaranteed or endorsed by the publisher.
