# Supplementary material for: Oxidative Stress Causes Masculinization of Genetically Female Medaka Without Elevating Cortisol
Source: Front Endocrinol (Lausanne). 2022 Jun 16;13:878286. doi: 10.3389/fendo.2022.878286 (PMC9272773; doi:10.3389/fendo.2022.878286)
Supplement: Supplementary file 1 [file Table_1.docx]

**Supplementary Table S1. Survival rates of the larvae during H_2_O_2_ and chemical treatments.**

**Supplementary Table S2. Body length and weight in the adult fish at 2 month age.**

**Supplementary Table S3. List of primers used in this study.**

**Supplementary Table S4. Expression stability of reference genes determined by RefFinder.**
